# Effect of Ultraviolet-C Light-Emitting Diode Treatment on Disinfection of Norovirus in Processing Water for Reuse of Brine Water

**DOI:** 10.3389/fmicb.2022.885413

**Published:** 2022-05-19

**Authors:** So-Ra Yoon, Sanghyun Ha, Boyeon Park, Ji-Su Yang, Yun-Mi Dang, Ji-Hyoung Ha

**Affiliations:** ^1^Hygienic Safety and Analysis Center, World Institute of Kimchi, Gwangju, South Korea; ^2^Eco-friendly Process Technology Research Group, World Institute of Kimchi, Gwangju, South Korea; ^3^Industrial Solution Research Group, World Institute of Kimchi, Gwangju, South Korea

**Keywords:** brine water, disinfection, inactivation kinetic model, norovirus, UVC LEDs

## Abstract

Processes in the food industry that use large amounts of water have been an important cause of waterborne disease outbreaks, as they expose individuals to risks for waterborne disease transmission. Developing technologies to ensure the hygiene and safety of food-processing steps is an urgent concern from an economic perspective. Furthermore, economic benefits can be derived if the processed water can be reused under microbiologically safe conditions. Among the major manufacturing processes in the kimchi industry, the brining process for salted kimchi cabbages requires a considerable amount of brine (approximately 2,000–2,500 l/1,000 kg of raw cabbage). The aim of this study was to establish virucidal conditions with ultraviolet-C light-emitting diodes (UVC LEDs) that can ensure the microbiological safety of brine water samples with various turbidities for reuse after disinfection. For quantitative analysis, first of all, magnetic bead separation (MBS) technique was used to capture and recover the human norovirus (HuNoV) virus particles; propidium monoazide (PMA) combined with RT-qPCR (PMA-RT-qPCR) was subsequently used to selectively detect infectious norovirus. Overall, as the turbidity of the brine water samples increased, the reduction in the HuNoV genogroup II genotype 4 (HuNoV GII.4) levels by UVC LED disinfection decreased. The derived inactivation rate constant (*k_inac_*) and inactivation curves (calculated using the log-linear model) were studied as a function of turbidity based on the exponential one-phase inactivation kinetics of HuNoV. Using an impeller system set at 100 rotations/min (rpm) with an eight-nephelometric turbidity unit (NTU) sample (the lowest turbidity studied), the *k_inact_* based on the levels of viral genomic RNA concentrations was approximately 2.15-fold higher than that observed without rotation (0 rpm). Moreover, the *k_inact_* increased 1.69-fold with a 56-NTU sample (the highest turbidity studied) when the impeller system was set at 100 rpm. UVC LED treatment decreased the HuNoV GII.4 population more effectively in conjunction with the impeller system (100 rpm) than without the impeller system. Our novel findings and model provide fundamental and scientific data that may help reuse brine water and ensure its microbiological safety through disinfection. Our study highlights the benefits of UVC LED treatment in successfully eliminating waterborne viruses in a prompt, resistance-reducing, and energy-efficient approach at the laboratory scale, which lays the foundation for future plant-scale studies of UVC LED-disinfection systems.

## Introduction

Approximately 97% of the water in Earth’s ecosystems is saline water, and only 1% is freshwater (World Health Organization, 2007). This freshwater scarcity faces increasing ecological and environmental pressure from climate change and population growth; thus, various studies have been performed with desalination plants to convert brine water to drinkable water and to supply it for reuse in the food industry (Shannon et al., 2008; Werber et al., 2016). Moreover, as the problem of environmental pollution becomes more serious, processing water discharged from industrial sites is emerging as a global problem (Yang et al., 2012).

In the kimchi-manufacturing industry, which uses a large amount of brine during cabbage brining, continuous efforts are being made to develop an eco-friendly process that can enable brine reuse. Universally, most brine waste discharged after brining cabbage during kimchi manufacturing is repeatedly used in the next brining process (Lee, 2008). Brine is reused 2.3 times on average, and the number of reuses increases by 3–5 times in the winter. The recycling of used brine can save the salt required for high-concentration brine (approximately 10–15%), which provides an economic advantage in terms of cost reduction (Kim et al., 2016).

In general, used water is related to the transmission of pathogenic viruses, including human norovirus (HuNoV), hepatitis A virus, and rotavirus, which can cause fatal waterborne diseases (Gil et al., 2009; Lynch et al., 2009; Sinha and Dutta, 2019). HuNoV are the most common cause of gastroenteritis and are responsible for at least 50% of all gastroenteritis outbreaks worldwide (Bartsch et al., 2016). In particular, HuNoV GII group is recognized as a major cause of acute gastroenteritis outbreaks, and sporadic illnesses caused by HuNoV GII.4 (Hartmann et al., 2015) and GII.2 (Mariita et al., 2022) have been reported. Infectious diseases caused by viral pathogens are mainly caused by cross-contamination from food surfaces during food-related procedures or by direct consumption/reuse of contaminated water (Brassard et al., 2011). Therefore, the levels of microbiological risk factors and changes in the number of coliforms, *Escherichia coli*, yeast, and mold have been universally considered as the control criteria indicators that most affect the frequency of brine reuse. In addition, as HuNoV has started to attract attention as a safety-management indicator for saltwater reuse, many efforts have been made to develop a quantitative detection method (Coudray-Meunier et al., 2013; Sangsanont et al., 2014; Lee et al., 2018).

The virucidal activity of ultraviolet-C (UVC, range 250–280 nm) radiation has been recognized as a reliable disinfection method for ensuring water safety (Hijnen et al., 2006; Chatterley and Linden, 2010). However, low- and medium-pressure mercury UV lamps can cause mercury pollution and have the disadvantages of a short bulb lifetime, low space utilization, sensitivity to temperature variations, and low energy efficiency (Vilhunen and Sillanpää, 2010). Recently, UVC light-emitting diodes (LEDs) have begun to replace conventional mercury UV lamps, with benefits that overcome the limitations of current technologies. UV LED treatment represents an emerging alternative disinfection treatment that is considered cost-effective, environmentally friendly, and sustainable (Korovin et al., 2015). In particular, compared with chemical disinfectant treatment, LED treatment ensures a safe water supply without generating disinfection by-products, odor, or unpleasant taste (Ibrahim et al., 2014). In addition, UVC LEDs exhibit the same virucidal control mechanism as the inactivation mechanism exhibited by conventional low- and medium-pressure mercury UV lamps (Bintsis et al., 2000; Yaun et al., 2004). Dimerization of pyrimidine-containing nucleic acid bases interferes with DNA replication and transcription, leading to cell death (Franz et al., 2009). The virucidal efficacy that can be expected from UVC LED-induced damage to nucleic acids (DNA or RNA) depends on the location of changes within the viral genome (Franz et al., 2009). Furthermore, to ensure the effectiveness of this UVC-LED sterilization effect, external factors that can affect fluorescence and wavelength (e.g., ultraviolet transmittance (UVT) and temperature and turbidity of fluid) must be taken into account (Severin et al., 1983; Mariita et al., 2022).

To the best of our knowledge, the results of numerous previous studies have demonstrated that UVC LED treatment can effectively inactivate pathogenic bacteria in processing water for its reuse, but additional studies of infectious viruses in processing water are required. The aim of this study was to investigate the efficacy of UVC LED-based virucidal activities to ensure the microbiological safety of brine processing water for reuse following disinfection. Therefore, we evaluated the application of UVC LED irradiation near 265 nm for disinfecting the human norovirus genogroup II genotype 4 (HuNoV GII.4), in brine processing water. The objectives of our study were to (1) investigate the profile of a used brine after brining kimchi cabbage and (2) study the virucidal effects of an effective UVC LED system on reused brine samples.

## Materials and Methods

### Viral Stocks

HuNoV GII.4 was obtained from the Catholic University of Korea (Seoul, South Korea). HuNoV GII.4 stock samples were diluted in RNase-free water (Qiagen, Hilden, Germany) and vortexed briefly. The viral supernatant suspension (6.88 ± 0.18 log_10_ genomic copies/mL) was stored in 500 μl aliquots at −80°C until use. For quantitative analysis, viral genomic RNA concentrations were determined by reverse transcriptase-quantitative polymerase chain reaction (RT-qPCR) analysis, following the protocol described in ISO 15216-1:2017 (ISO 15216-1:2017, 2017), as previously described by Lee et al. (2018). The entire process of preparing the viral stocks was carried out with strict adherence to biosafety considerations (Mariita et al., 2022). The entire process of preparing the viral stocks was carried out with strict adherence to biosafety considerations (Mariita et al., 2022).

### Used Brine Source and Artificial Contamination

Brine was collected daily for 5 days from a kimchi-manufacturing company located in Gwangju, Korea. The microbial and physicochemical properties of the collected brine samples were analyzed and their characteristics were profiled. All microbial and physicochemical parameters were selected according to standard methods for examining brine water (APHA, 2019). The brine water profiling was based on eight parameters, namely the pH, nephelometric turbidity units (NTU), chemical oxygen demand (COD), biochemical oxygen demand (BOD), dissolved oxygen (DO), total coliform (TC) level, *Escherichia coli* level, and HuNoV level. The salinity of all experimental brine water samples was 14 ± 1.1%. Samples were denoted according to different NTUs (8, 16, 24, 32, 40, 48, and 56 NTUs), which corresponded to the number of reuses. KimchiTown Co. (Gwangju, Korea) kindly provided used brine water samples from seven groups (numbered according to their consecutive number of reuses; 0, 1st, 2nd, 3rd, 4th, 5th, and 6th), which had been previously evaluated as negative for HuNoV GII.4 based on the ISO 15216-1:2017 method. Each experimental setup was designated as one experimental batch, and 300 ml of brine water samples artificially inoculated with HuNoV GII.4 was prepared for each experimental batch. HuNoV GII.4 was resuspended in 300 ml of each brine water sample (denoted as 0, 8, 16, 24, 32, 40, 48, and 56 NTU samples), and 1 ml stock suspension of HuNoV (containing 6.88 ± 0.18 genomic copies) was inoculated individually into 300 ml of each batch (used brine water samples), corresponding to approximately 6.90 log_10_ genomic copies/batch of HuNoV GII.4.

### Experimental Setup and UVC LED Treatment

**Figure 1:**
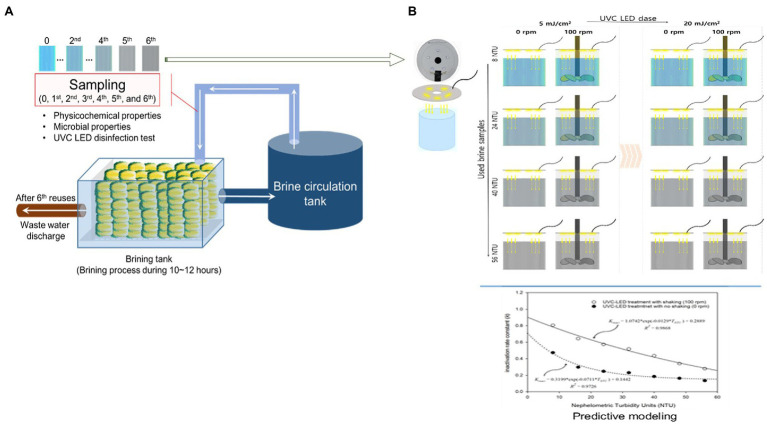
Schematic diagram of **(A)** manufacturing process for brining cabbage and **(B)** the UVC LED-treatment in conjunction with the impeller system for disinfection of used brine water samples.

In this study, UVC LED irradiation (peak wavelength of approximately 275 nm) treatment was conducted to inactivate HuNoV GII.4 in used brine water. Electronic printed circuit boards (PCBs), based on UVC LED modules (SeoulViosys Co., Seoul, Korea) corresponding to the peak wavelength (approximately 275 nm), were utilized (Figure 1). UV LEDs with emissions at 275 nm and an optical power output of 60 mW at a current of 30 mA and voltage of 35 V were used. For the UVC LED treatment, the PCB connected to the LED-UVC was fixed on the top of the frame, and each 300 ml brine water sample was placed 5 cm away. The brine water samples that were inoculated with HuNoV were divided into two groups. One group was subjected to UVC LED treatment without stirring. The other group was treated with a UVC LED while stirring at 100 rotations/min (rpm) using an impeller (Supplementary Figure S1). The intensities of the UVC LED modules were measured with a spectrometer (AvaSpec-ULS2048-USB2-UA-50; Avantes, Apeldoorn, Netherlands). Using this experimental setup, the modified irradiance intensity of the UVC LED was 20.27 ± 1.17 μW/cm^2^. The treatment dosages were determined based on preliminary experiments. The inoculated brine water samples were treated at dosages of 0, 5, 10, 15, and 20 mJ/cm^2^ for all inactivation treatments.

### Viral Population Enumeration

#### RT-qPCR Analysis of the HuNoV Levels

To capture and recover HuNoV viral particles from each batch after UVC LED disinfection, we performed magnetic bead separation (MBS) using commercial Viro-adembeads (Ademtech, Pessac, France). With the MBS method, to concentrate whole viral HuNoV GII.4 particles from each batch, 75 μl of anionic polymer-coated magnetic bead solution (final concentration: 20 mg/ml) and 50 ml of the viral particle suspension were mixed in a 50-mL conical tube and agitated for 1 h at 20 ± 2°C. This step was repeated 10 times for each 500 ml batch. A LifeSep MBS stand (Sigma-Aldrich) was used to separate the supernatant and magnetic beads from the viral particles captured from the suspension. The magnetic beads with captured HuNoV viral particles were resuspended in 420 μl of RNase-free water. For MBS-based viability determinations, real-time RT-qPCR was performed after pretreatment with propidium monoazide (PMA; MBS/PMA/RT-qPCR), where 420 μl of the captured HuNoV viral particle suspension was mixed with 100 μl RNase-free water containing 200 μM PMA (Lee et al., 2018). Using the intercalating-dye method, Lee et al. (2018) verified that 200 μM PMA (Biotium, Hayward, CA, United States) was optimal for quantifying intact HuNoV viral particles while minimizing particle loss. The viral-particle suspensions were incubated in the dark at 5°C for 15 min to allow time for dye binding. Photoactivation between viral nucleic acid and PMA was induced with a high-power LED light (45 W lamp) at 460 nm wavelength for 20 min at 5°C, using the PhAST Blue Photoactivation System (GenIUL, Barcelona, Spain). Viral RNA extraction and quantitative RT-qPCR were conducted following the protocol detailed in ISO 15216-1:2017 (ISO 15216-1:2017, 2017). The probe and primers (Bioneer Inc. Daejeon, Korea) used in this study were reported previously (Lee et al., 2018).

### Mathematical Modeling

To analyze the inactivation kinetics of UVC LED treatment against HuNoV GII.4 in used brine water following irradiation, the resulting data were analyzed using the GInaFIT software (version 1.6), a freeware tool (Geeraerd et al., 2005). The inactivation plots fit well with the log-linear model (Eq. 1) and the inactivation rate constant (*k_inact_*) was calculated (Eq. 2).


(Eq. 1) $$Log_{10}R=log_{10}\left(\frac{N_{0}}{N}\right)=\frac{k_{max}}{ln\left(10\right)}\times D$$

(Eq. 2) $$k_{\max}=k_{inact}\times \ln\left(10\right)$$

where *Log_10_R* is the infectivity reduction on a log_10_ scale; *N* is the infectious HuNoV titer (log_10_ genomic copies/μL) after UVC LED treatment; *No* is the initial infectious viral titer (log_10_ genomic copies/μL); *k_inact_* is the pseudo-first-order inactivation rate constant at a given inactivation dose (mJ/cm^2^). The maximum inactivation rate constant (*k_max_*) was calculated using a specific dose (D) of UVC LED irradiation, expressed as the mJ/cm^2^.

The induced *k_inact_* was calculated as a function of turbidity using SigmaPlot (version 14, San Jose, CA, USA). The interrelationship between the sample turbidity and inactivation rate was used to illustrate the exponential one-phase inactivation kinetics of HuNoV (Eq. 3). The developed regression model was combined with a logistic linear model to predict HuNoV inactivation as a function of turbidity (NTU) and the UVC LED irradiation dose (D).


(Eq. 3) $$k=\left(k_{0}-a\right)\times exp\left(-b\times T_{TNU}\right)+a$$

where *T_NTU_* is the NTU of a brine water sample, *k*_0_ is the *k_inact_* at an NTU of 0, *a* is the *k* value at infinite turbidity, and *b* is the rate constant. From the above regression equation, the following equations were derived to predict HuNoV inactivation as a function of the T_NTU_ and UVC LED D values.

For HuNoV GII.4, with UVC LED irradiation, but without shaking (0 rpm),


$$Log_{10}\left(\frac{N_{0}}{N}\right)=\left[0.3199.exp\left(-0.0711.T_{NTU}\right)+0.1442\right]\times D$$

For HuNoV GII.4 with the UVC LED irradiation and shaking (100 rpm),


$$Log_{10}\left(\frac{N_{0}}{N}\right)=\left[1.0742.exp\left(-0.0129.T_{NTU}\right)+0.2889\right]\times D$$

### Validation of the Predictive Model

We investigated whether the developed model could predict HuNoV GII.4 inactivation based on turbidities other than those used experimentally. The turbidity of the brine processing water samples, which were provided from KimchiTown Co. (Gwangju, Korea), for further experiments was 28 and 36 NTU. The turbidities were within the range previously used to develop the model. The accuracy factor (*A_f_*) and bias factor (*B_f_*) were used for validation purposes and were calculated using the following equations:


(Eq. 4) $$A_{f}=10^{\left[\;\frac{\sum |log\left(\frac{Y_{model}}{Y_{obs}}\right)|}{n}\right]}$$

(Eq. 5) $$B_{f}=10^{\left[\;\frac{\sum log\left(\frac{Y_{model}}{Y_{obs}}\right)\;}{n}\right]}$$

where *Y_model_* is the modeled value, *Y_obs_* is the observed value, and n is the number of experimental replicates. The root-mean-square error (RMSE) of the mathematical kinetic model prediction with respect to the estimated variable *Y_model_* was defined as the square root of the mean squared error (Eq. 6):


(Eq. 6) $$\mathrm{RMSE}=\sqrt{\frac{\sum \frac{n}{i=1}{\left(Y_{obs,i}-Y_{model,\;i}\right)}^{2}}{n-k}}$$

where *Y_model_* is the modeled value, *Y_obs_* is the observed value, *n* is the number of observations, and *k* is the number of estimated parameters in the model. The residual sum of squares (RSS) was determined as the sum of the squared distances between the predicted values and empirical data:


(Eq. 7) $$\mathrm{RSS}=\overset{n}{\underset{i=1}{\sum }}{\left(Y_{obs,i}-Y_{model,i}\right)}^{2}\;\;\;\;\;\;\;$$

where *Y_model_* is the modeled value, and *Y_obs_* is the observed value. The Akaike information criterion (AIC) formula (Eq. 8) was used for the sum of squares optimization:


(Eq. 8) $$AIC=n\times \ln\left(\frac{ss}{n}\right)+2\times k$$

where *n* is the number of observations, *k* is the number of estimated parameters in the model, and *SS* is the sum of the squares.

### Statistical Analysis

Duplicate samples were tested for each brine water sample, and the experiments were repeated in triplicate. For statistical analysis, one-way analysis of variance was used to compare differences among mean values using the SPSS Statistics software (version 19, IBM Corp., Chicago, IL, United States). The threshold for statistical significance was set at *p* < 0.05. The experimental results were determined as log_10_ genomic copies/μL, and regression analysis was performed using the SigmaPlot software (version 14.0).

## Results and Discussion

### Recovery Rate of HuNoV GII.4

To evaluate the efficiency of MBS/PMA/RT-qPCR, we first investigated the recovery rates from processing water artificially inoculated with HuNoV GII.4. The mean recovery rates of HuNoV GII.4 between the RT-qPCR method alone, as a negative control, and PMA/MBS-qRT-PCR were compared. The mean recovery quantities of HuNoV determined using the MBS/RT-qPCR and MBS/PMA/RT-qPCR assay were 91.74 ± 1.3% and 88.4 ± 2.5% for NoV (Supplementary Figure S2). The use of 200 μM PMA had no significant effect on the quantitation of HuNoV GII.4 virus particles (*p* > 0.05).

### Influence of Turbidity on Pathogen Inactivation by UVC LED Irradiation

The microbial and physicochemical properties of the collected brine samples were analyzed, as shown in Table 1. As the frequency of brine use increased, the pH and DO values decreased significantly, whereas the turbidity, BOD, and COD increased significantly. As the number of uses increased by one, the turbidity increased proportionally. In terms of microbiological changes, *E. coli* and HuNoV were not detected in any of the samples, and TC was detected at a level of approximately 2 log_10_ colony-forming units (CFUs)/mL in brine water used consecutively for five or six times.

**Table 1:** Microbial and physicochemical characteristics of brine water.

NO^1^	pH	NTU^2^	DO^3^	BOD^4^	COD^5^	TC^6^	*E. coli* ^7^	HuNoV
0	6.95 ± 0.07	7.60 ± 0.84	8.55 ± 0.07	10.25 ± 1.21	9.20 ± 0.57	ND^8^	ND	ND
1^st^	5.65 ± 0.01	15.85 ± 1.04	0.70 ± 0.07	202.75 ± 15.21	20.80 ± 0.81	ND	ND	ND
2^nd^	5.30 ± 0.07	27.69 ± 3.81	0.20 ± 0.07	322.50 ± 28.99	24.80 ± 0.41	ND	ND	ND
3^rd^	5.20 ± 0.21	31.22 ± 2.01	0.15 ± 0.05	502.50 ± 6.36	27.10 ± 0.59	ND	ND	ND
4^th^	5.15 ± 0.05	39.52 ± 1.23	0.10 ± 0.00	614.50 ± 29.71	29.50 ± 0.82	ND	ND	ND
5^th^	5.12 ± 0.07	47.64 ± 0.46	0.08 ± 0.00	708.34 ± 15.68	30.01 ± 0.55	1.18 ± 0.46	ND	ND
6^th^	5.10 ± 0.14	57.02 ± 0.28	0.05 ± 0.00	789.75 ± 23.33	31.90 ± 0.79	1.34 ± 0.42	ND	ND

^1^Number of reuses; ^2^nephelometric turbidity units; ^3^dissolved oxygen (mg/L); ^4^biochemical oxygen demand (mg/L); ^5^chemical oxygen demand (mg/L); ^6^total coliform (log_10_ CFUs/mL); ^7^Escherichia coli; and ^8^not detected.

The virucidal effect of UVC LED irradiation was affected by increased turbidity when the impeller system was not used to stir the samples (0 rpm; Table 2). Regarding the virucidal effect of UVC LED irradiation in used brine water samples, the HuNoV concentrations after UVC LED treatment at 5 or 10 mJ/cm^2^ showed maximum decreases of 3.44 and 3.76 log_10_ genomic copies/μL, respectively, in 8 NTU water samples. Overall, as the brine water turbidity increased, the reduction in HuNoV caused by UVC LED treatment decreased. This was especially true at the maximum turbidity (56 NTUs), where the water samples contained 0.37 or 0.51 log_10_ genomic copies/μL after exposure to 5 or 10 mJ/cm^2^ UVC LED, respectively.

**Table 2:** Changes in the populations of HuNoV GII.4 after UVC LED treatment in re-used brine water samples with different turbidities.

Impeller system (rpm)	NO^1^	NTU^2^	UVC LED dose (mJ/cm^2^)				
			0	5	10	15	20
0	0	8	5.61 ± 0.22	2.17 ± 0.29	1.85 ± 0.19	ND^3^	ND
	1^st^	16	5.65 ± 0.11	3.28 ± 0.31	2.97 ± 0.09	ND	ND
	2^nd^	24	5.71 ± 0.21	3.56 ± 0.19	3.23 ± 0.13	ND	ND
	3^rd^	32	5.58 ± 0.23	4.01 ± 0.22	3.95 ± 0.09	1.88 ± 0.32	ND
	4^th^	40	5.59 ± 0.27	4.32 ± 0.15	4.12 ± 0.05	2.49 ± 0.18	1.98 ± 0.34
	5^th^	48	5.67 ± 0.34	4.93 ± 0.10	4.59 ± 0.12	2.86 ± 0.28	2.47 ± 0.22
	6^th^	56	5.56 ± 0.22	5.19 ± 0.15	5.05 ± 0.16	3.16 ± 0.14	2.69 ± 0.18
100	0	8	5.75 ± 0.25	1.72 ± 0.11	ND	ND	ND
	1^st^	16	5.67 ± 0.22	2.45 ± 0.25	ND	ND	ND
	2^nd^	24	5.64 ± 0.38	2.77 ± 0.45	ND	ND	ND
	3^rd^	32	5.81 ± 0.23	2.91 ± 0.32	ND	ND	ND
	4^th^	40	5.66 ± 0.41	3.12 ± 0.83	1.29 ± 0.27	ND	ND
	5^th^	48	5.75 ± 0.29	3.19 ± 0.54	1.71 ± 0.10	1.54 ± 0.18	ND
	6^th^	56	5.73 ± 0.22	3.34 ± 0.65	1.56 ± 0.04	1.31 ± 0.15	ND

^1^Number of reuses; ^2^nephelometric turbidity units; and ^3^not detected.

We anticipated that suspended solids could interfere with the virucidal activity by reducing the transmittance of UVC LED irradiation. Surviving populations of HuNoV GII.4 (log_10_ genomic copies/μL) after 15 or 20 mJ/cm^2^ UVC LED treatment were not detected in the 8, 16, and 24 NTU brine water samples, whereas the HuNoV GII.4 levels in 56 NTU brine water samples were detected by 2.40 (at 15 mJ/cm^2^) and 2.87 (at 20 mJ/cm^2^) log_10_ genomic copies/μL. Interestingly, the virucidal activities of UVC LED treatment were higher when using the impeller system (set at 100 rpm) than when the impeller system was not used (Table 2). In particular, 10 mJ/cm^2^ UVC LED treatment inactivated HuNoV GII.4 in brine water samples at an NTU of up to 32. Furthermore, HuNoV GII.4 in the 56 NTU samples was not detected after UVC LED treatment at ≥20 mJ/cm^2^ with impeller system set at 100 rpm. Overall, the HuNoV values were reduced significantly more by UVC LED treatment with the impeller system (100 rpm) than without the impeller system, with all tested brine water samples. UVC LED disinfection can be sensitive to turbidity (Gullian et al., 2012; Carré et al., 2018). Gullian et al. (2012) demonstrated that an increase in turbidity in water samples from 10 to 16 NTU resulted in a 60% decrease in the antimicrobial activities of UVC LED treatment. Carré et al. (2018) verified that the antimicrobial activity of UVC LED irradiation was maintained without significant changes when the turbidity was below 5 NTU.

Moreover, the exposure frequency of disinfecting UVC LED light sources and UVT of the water sources also affect the virucidal efficacy. As the turbidity increases and UVT decreases, there is a limit to how deep a UVC LED light source can penetrate water, which is an important factor in sterilization. In particular, understanding the UVT of the processing water source is an important factor to ensure that the treated UVC LED dose is sufficient to inactivate pathogenic viral particles (Jarvis et al., 2019). In this regard, our experimental results demonstrate that the water stirring system significantly improves the UVC LED-disinfection levels caused by turbidity. UVC LED treatment is an energy-based disinfection process, where the microbial inactivation efficiency is determined by various parameters including the treated environmental condition and the applied dose of the UV light source (Gidari et al., 2021). Theoretically, the dose (mJ/cm^2^) of UVC LEDs is determined by the exposure time (s) multiplied by the intensity delivered to microbial cells (mW/cm^2^). Therefore, for UVC LED treatment-induced inactivation, the UV dose (rather than the exposure time) is the most precise measure of the efficacy of disinfection (Nyangaresi et al., 2018). Thus, regarding virucidal mechanisms based on UVC LED treatments, fatal damage to microbial cells occurs over a UVC LED range because the intercellular components of microbes (such as RNA and capsid proteins) can sensitively absorb UVC photons. Damage to viral proteins and/or viral nucleic acids is well known as a general mechanism of virus inactivation by UVC LED disinfection (Wan et al., 2020). For example, a UVC LED light source can disintegrate the viral capsid protein of murine norovirus-1 (Park et al., 2016). UVC LED treatment caused oxidative damage to the viral capsid protein, which was linked to reduced infectivity with HuNoV (Sano et al., 2010; Tanaka et al., 2018) and bacteriophage MS2 (Rule Wigginton et al., 2010). Poliovirus inactivation has been demonstrated to occur secondarily to viral protein–genome crosslinking induced by UVC treatment (Wetz and Habermehl, 1982). UVC has been shown to inactivate severe acute respiratory syndrome coronavirus 2 by destroying viral nucleic acids (Gidari et al., 2021).

Viruses are frequently classified as having single-stranded DNA, double-stranded DNA, single-stranded RNA, or double-stranded RNA (Kowalski, 2009). Such viruses show different susceptibilities to UV irradiation; therefore, virucidal effects vary depending on the type of genome involved. Generally, single-stranded viruses, including HuNoVs, are more sensitive to UVC LED treatment because of a lack of redundant genetic information in the second strand, which enables double-stranded viruses to repair the damage (Tseng and Li, 2005). In contrast, non-enveloped viruses (including HuNoVs) typically show more UVC resistance than enveloped viruses because lipids and capsid proteins in an envelope can be destroyed more easily than other parts of viruses (Perlmutter and Hagan, 2015). In general, inactivation of pathogenic microorganisms occurs in the UV wavelength range of 250–280 nm, and the maximum effect has been confirmed around 265 nm (Chatterley and Linden, 2010). Therefore, previous studies reported that relative germicidal effectiveness of UV-C energy based on microbial DNA damage depends on various wavelengths (Bintsis et al., 2000; Hijnen et al., 2006). Moreover, many factors influence the germicidal effect of UVC LED, and the main factor is the operating temperature of LED chips. As electric energy is applied to the LED, it is converted into heat and light. The heat generated increases the LED junction temperature, reducing the light output, which is a major factor in the sterilization effect. Therefore, it is important to properly manage the operating temperature generated by the UVC LED irradiation (Arques-Orobon et al., 2020).

For quantitative analysis, we performed molecular biology assays, unlike the cell culture technology used by other researchers. The overestimation of infectious viruses after disinfection treatment and the inability to differentiate between inactivated and infectious viruses is considered a main disadvantage of RT-qPCR (Karim et al., 2015). Therefore, the developed method, such as the MBS/PMA/RT-qPCR assay, could allow for more precise quantification of potentially infectious viral particles in environmental samples after disinfection (Lee et al., 2018). In particular, Karim et al. (2015) demonstrated that pretreatment with the intercalating dye PMA combined with RT-qPCR assay could potentially be used with all non-culturable or fastidious viruses, although specific conditions of inactivation must be investigated.

### Kinetics of the Influence of Turbidity on HuNoV GII.4 Inactivation by UVC LED Irradiation

**Figure 2:**
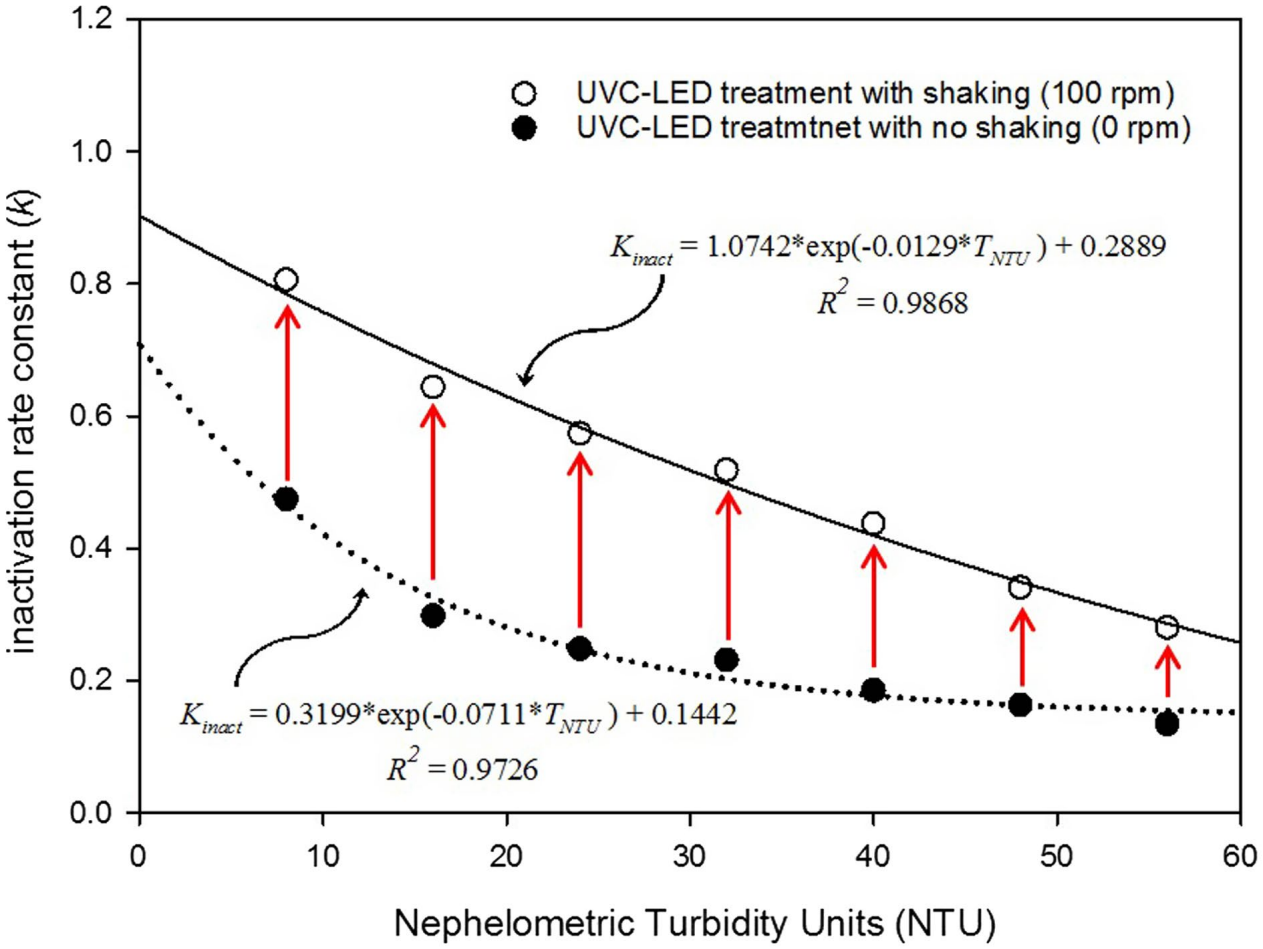
Relationship between the inactivation rate constant (*k_inact_*) values and nephelometric turbidity units.

With the inactivation regression model of HuNoVs treated with UVC LED, we observed that increased turbidity in the used brine water samples decreased the virucidal effect of UVC LED treatment. The most noteworthy and interesting findings of this study are presented in Figure 2. Figure 2 presents the inactivation curves, which were drawn using a log-linear model. We confirmed that operating the impeller system during UVC LED treatment increased the virucidal effects obtained when disinfecting turbid samples. Our results indicated that the *k_inact_* increased by approximately 2.15-fold when using the impeller system at 100 rpm with the 8 NTU samples (lowest turbidity studied) and that the *k_inact_* increased by 1.69-fold even in the 56 NTU samples (highest turbidity studied). Importantly, the test group exposed to UVC LED with the impeller system set at 100 rpm showed a tendency toward a delayed decrease in the *k_inact_* (Figure 2). Furthermore, the developed model can be used to predict the virucidal effect of turbidity-dependent UVC LED disinfection with brine water samples. In this regard, the developed model can be used to determine the processing conditions when considering the titers of target viruses, the water-sample turbidity, and the UVC LED dose. Universally, the *k_inact_* has been mostly used as a mathematical parameter to explain pathogenic microorganism inactivation by UVC disinfection and has also been applied to compare the resistance of pathogenic microorganisms to UVC disinfection (Goldman and Travisano, 2011; Viana et al., 2013). In cases where the resistance to UVC LED treatment is high, the *k_inact_* appears to be low, and in general, *k_inact_* values appear to be significantly lower for infectious viruses than for pathogenic bacteria (Weiss and Horzinek, 1986).

### Validation of the Developed Kinetics to Predict Pathogen Inactivation by the UVC LED Irradiation

The interrelationship between the *k_inact_* and brine water sample turbidity was determined mathematically by regression analysis, and the fitness of the regression models agreed well with the exponential one-phase decay model (Figure 2). The five model-selection criteria used to determine the correspondence between the two models are presented in Table 3. The better the fit of the developed model, the lower the RMSE, RSS, and AIC. Thus, the lowest value obtained from the model-selection criteria implied a reliable predictive model.

**Table 3:** Comparison of the predictive models in terms of the model-selection criteria including the accuracy factor, bias factor, RMSE, RSS, and AIC based on goodness of fit.

RPM	NTU^1^	UVC LED (mJ/cm^2^)	*Af* ^2^	*Bf* ^3^	RMSE^4^	RSS^5^	AIC^6^
0	28	5	1.31	0.77	0.0657	5.237	7.357
		15	1.27	0.87	0.0493	3.246	6.848
	36	5	1.23	0.82	0.0486	4.158	6.958
		15	1.30	0.74	0.0442	1.174	5.124
100	28	5	1.08	0.95	0.0254	0.967	1.028
		15	1.14	0.91	0.0279	0.687	1.988
	36	5	1.08	0.88	0.0292	0.785	1.289
		15	1.12	1.07	0.0177	0.987	2.358

^1^Nephelometric turbidity units; ^2^accuracy factor; ^3^bias factor; ^4^ root-mean-square error; ^5^residual sum of squares; and ^6^Akaike information criterion.

With the model developed using the UVC LED and impeller system, the model-selection criteria (including the RMSE, RSS, and AIC based on goodness of fit) demonstrated better goodness-of-fit than the model without the impeller system (Table 3). Both models provided suitable fits based on small RMSE, RSS, and AIC values with both types of UVC LED treatment (i.e., with/without an impeller system). In terms of the *A_f_* and *B_f_* values, an *A_f_* and *B_f_* of 1 indicates an absolute agreement between the observations and predictions. An *A_f_* value above 1 represents a less accurate average estimate, and generally the farther the *B_f_* value is from 1, the more a model underpredicts or overpredicts the experimental data. The virucidal effects observed using UVC LED treatment without the impeller system were not a good fit with the predicted values, with *A_f_* values ranging from 1.23 to 1.31 and *B_f_* values ranging from 0.74 to 0.87. The data obtained after UVC LED treatment with an impeller system showed model fittings, with *A_f_* values ranging from 1.08 to 1.12 and *B_f_* values ranging from 0.88 to 1.07. These statistical analysis values demonstrate that UVC LED treatment conditions should be approached conservatively to inactivate HuNoV GII.4. Comprehensively, it is necessary to consider several additional factors because our statistical analysis of the virucidal effects against HuNoV was only based on water sample turbidities. For instance, various viral pathogens are necessary for additional evaluation because of their fundamental variations. In addition, it is necessary to examine correlations between changes in the virucidal effect and other parameters (beyond the NTU) such as the temperature, BOD, COD, and microbacterial communities, and it is necessary to determine specific conditions for the most efficient UVC LED treatment. In conclusion, a statistical technique using predictive modeling can predict the sterilization effect of UVC LED treatment as an approach suitable for safe disinfection of brine for reuse in the future.

## Conclusion

In this study, we studied UVC LED-based HuNoV GII.4 inactivation in brine water samples and enhancement of the virucidal effect by including an impeller system. The influence of turbidity on the virucidal effect against HuNoV was mathematically analyzed based on the *k_inact_* to compare the inactivation rate by UVC LED irradiation. UVC LED treatment with the impeller system set at 100 rpm reduced the HuNoV GII.4 population more effectively than UVC LED treatment without the impeller system, and it was less affected by increased sample turbidity. Moreover, the developed model was validated with brine water samples. In conclusion, our novel findings and developed model could provide fundamental and scientific data for reusing brine and assure the microbiological safety of brine processing water for reuse after disinfection.

## Data Availability Statement

The original contributions presented in the study are included in the article/supplementary material, further inquiries can be directed to the corresponding author.

## Author Contributions

SR-Y and SH were responsible for conducting the experiments, assessing the experimental data, and writing the first draft of the manuscript. BP, J-SY, and Y-MD were responsible for assessing the experimental data. J-HH was responsible for coordinating the data, designing the experiments, analyzing and interpreting the data, and writing, revising, and finalizing the manuscript. All authors read and approved the final manuscript.

## Funding

This research was supported by a grant from the World Institute of Kimchi (KE2202-2) and was funded by the Ministry of Science and ICT, Republic of Korea.

## Conflict of Interest

The authors declare that the research was conducted in the absence of any commercial or financial relationships that could be construed as a potential conflict of interest.

## Publisher’s Note

All claims expressed in this article are solely those of the authors and do not necessarily represent those of their affiliated organizations, or those of the publisher, the editors and the reviewers. Any product that may be evaluated in this article, or claim that may be made by its manufacturer, is not guaranteed or endorsed by the publisher.
