# Supplementary material for: Effect of Ultraviolet-C Light-Emitting Diode Treatment on Disinfection of Norovirus in Processing Water for Reuse of Brine Water
Source: Front Microbiol. 2022 May 19;13:885413. doi: 10.3389/fmicb.2022.885413 (PMC9161207; doi:10.3389/fmicb.2022.885413)
Supplement: Supplementary file 1 [file Table_1.DOCX]

Effect of ultraviolet-C light emitting diode treatment to the norovirus disinfect processing water for brine water reuse

So-Ra Yoon^a†^, Sanghyun Ha^a†^, Boyeon Park^b^, Ji-Su Yang^c^, Yun-Mi Dang^a^, Ji-Hyoung Ha^a*^

^a^Hygienic Safety and Analysis Center, World Institute of Kimchi, Gwangju 61755, Korea

^b^Eco-friendly Process Technology Research Group, World Institute of Kimchi, Gwangju 61755, Korea

^c^Industrial Solution Research Group, World Institute of Kimchi, Gwangju 61755, Korea


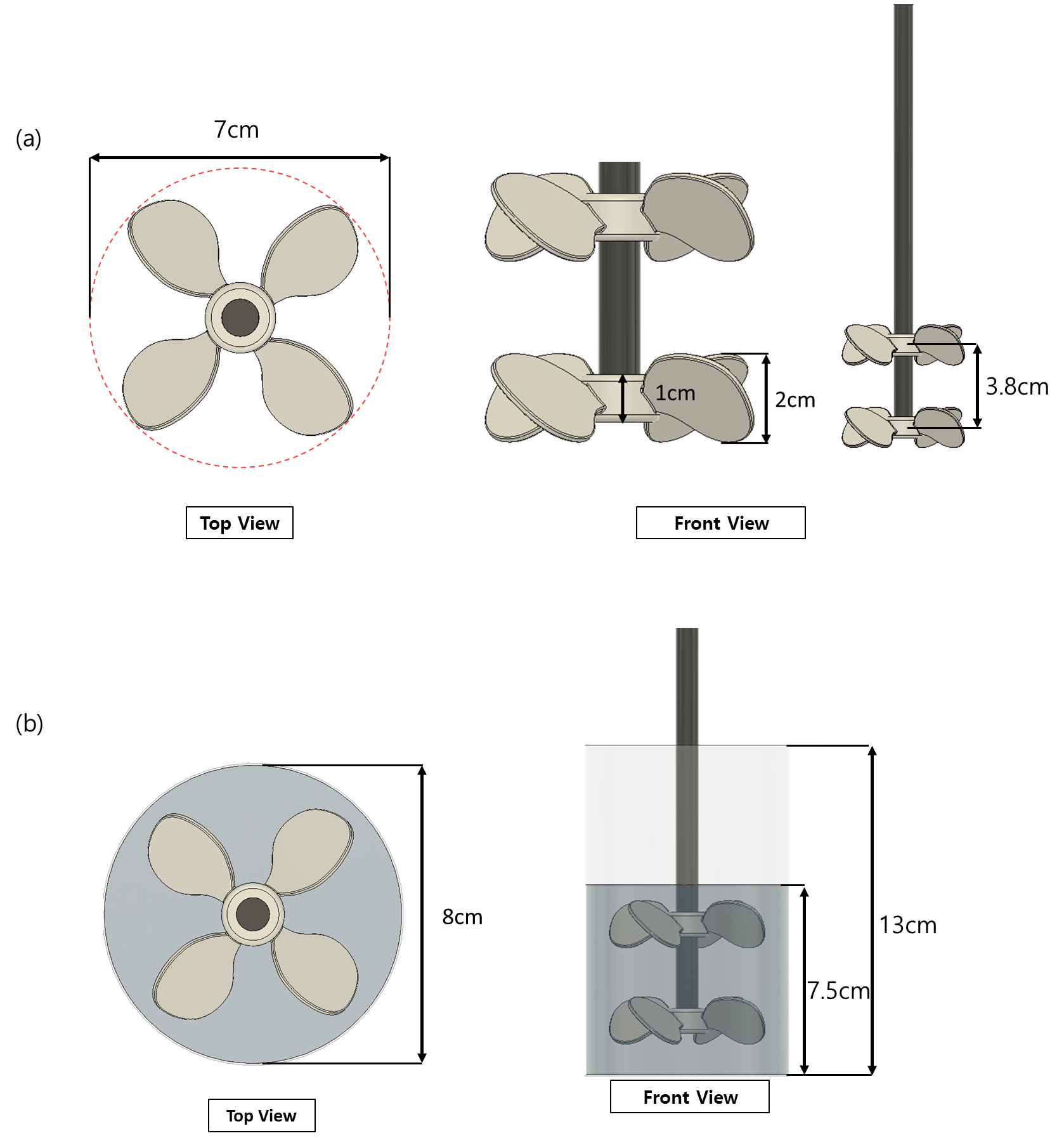


Fig. S1. A schematic image of the impeller system used for brine processing water. (a) with the double 4-blade impeller and (b) the blade impeller in brine processing water.


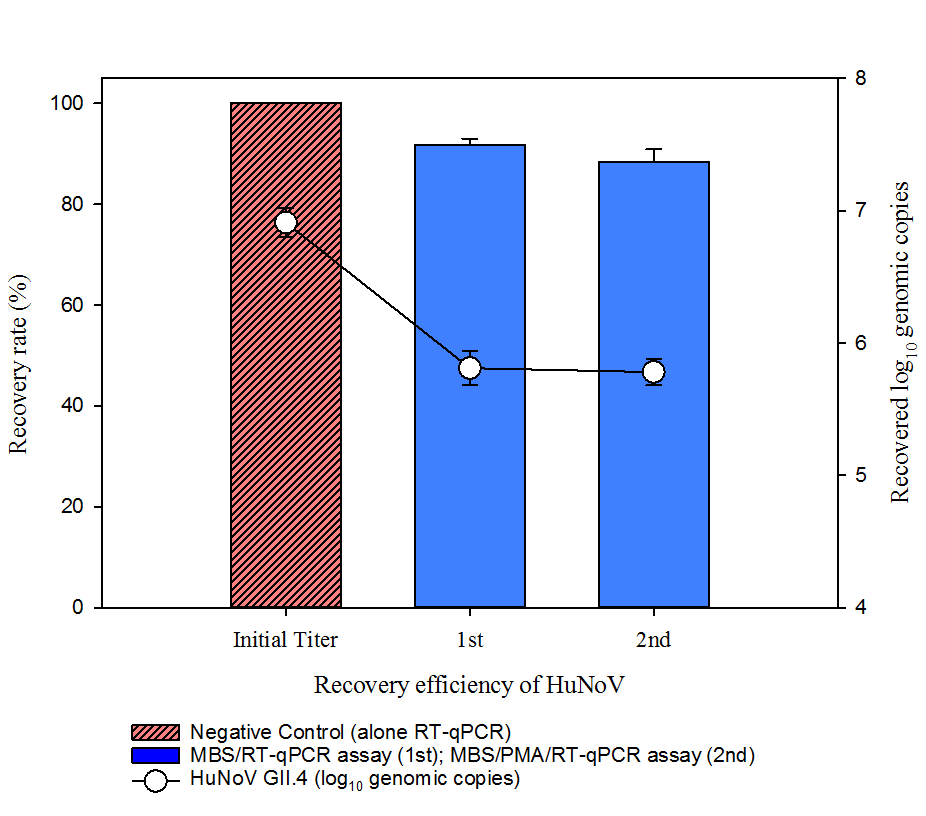


Fig. S2. The comparison of recovery efficiency of human norovirus GII.4 from brine processing water using the alone RT-qPCR assay as negative control and the MBS/PMA/RT-qPCR assay.
